# Acute Acalculous Cholecystitis Complicating Typhoid Fever: A Case Report

**DOI:** 10.7759/cureus.95524

**Published:** 2025-10-27

**Authors:** Fahmida Miah, Zohaib Khan, Muhammad Ali

**Affiliations:** 1 Acute Medicine, Walsall Healthcare NHS Trust, Walsall, GBR

**Keywords:** acute acalculous cholecystitis (aac), enteric fever (typhoid fever), fever in returning traveller, salmonella typhii, tropical infectious diseases

## Abstract

Typhoid fever continues to represent a significant public health concern in the developing world. With the rise in global travel, increasing population diversity, and the emergence of multidrug-resistant strains, clinicians must remain vigilant to both the disease and its potential complications. Among these, acalculous cholecystitis is an uncommon but clinically important complication. While the majority of reported cases in the literature involve paediatric patients, reports in adults remain scarce. We present the case of a 25-year-old traveler who developed acute acalculous cholecystitis as a complication of typhoid fever, a rare occurrence in adults that underscores the need for clinical vigilance.

## Introduction

Typhoid fever, also known as enteric fever, is a major systemic infection and a significant public health burden in the developing world. It is caused by the ingestion of *Salmonella enterica* serovar Typhimurium (*Salmonella* Typhi), a gram-negative bacillus. The term enteric fever also encompasses infections due to *Salmonella* Paratyphi A and B, which typically result in a clinically similar but often less severe presentation [1]. Transmission occurs via the faecal-oral route, most commonly through ingestion of contaminated water or food. According to the World Health Organization (WHO), typhoid fever accounts for an estimated 11 million to 20 million cases per year globally and approximately 140,000 deaths annually [2].

The onset of symptoms typically occurs following an incubation period of seven to 14 days [3]. The characteristic clinical feature is a gradually progressive fever, often rising in a stepwise manner and plateauing at 39-40°C. Additional common manifestations include abdominal pain, diarrhoea, nausea, headache, and cough [3]. A detailed clinical history, in combination with the presence of one or more gastrointestinal symptoms and a progressively developing fever, is instrumental in guiding the diagnosis.

The incidence of typhoid fever is extremely low in the developed world (less than one case per 100,000 individuals per year), but it is the most common bacterial cause of fever in returning travellers from endemic areas [4]. Complications occur in approximately 10-15% of patients hospitalised with typhoid fever, with encephalopathy, gastrointestinal bleeding, nephritis, and hepatitis being the most frequently reported [4]. Acalculous cholecystitis, however, represents one of the rarest complications. Acalculous cholecystitis involves acute inflammation of the gallbladder in the absence of gallstones, accounting for 5-10% of all cases of acute cholecystitis [5]. If left untreated this can lead to major surgical complications such as gallbladder perforation or gangrenous necrosis [5].

Although acute acalculous cholecystitis secondary to typhoid fever has previously been reported in paediatric patients from developing countries [6], reports in adult patients remain rare. This case report describes typhoid fever complicated by acalculous cholecystitis in an adult Pakistani male patient, highlighting a rare presentation and the need for heightened clinical awareness in adults.

## Case presentation

A 25-year-old male patient presented to the hospital with a 13-day history of cough, fever, diarrhoea, and vomiting. He had no significant past medical history. The patient had recently immigrated to the United Kingdom (UK) from Pakistan. He developed a dry cough on the first day after his arrival in the UK, followed by fever on the subsequent day. Four days later, he experienced diarrhoea, which was subsequently followed by vomiting.

On admission, the patient was pyrexial (39.5 °C), tachycardic (120 beats per minute), and normotensive (120/70 mmHg). Physical examination revealed mild, generalized tenderness in the right hypochondrium, with the absence of guarding. Laboratory investigations demonstrated an initial leukocytosis and abnormal liver function tests, including elevated bilirubin and gamma-glutamyl transferase (GGT) levels (Table 1).

**Table 1:** Results of blood tests during admission

	Reference range (Units)	Day of admission (Day 0)	Day 2	Day 4
Haemoglobin	130-180 (g/L)	140	114	115
White Cell Count	4-11 (x10^9^/L)	14.8	2.9	2.3
Platelet Count	150-450 (x10^9^/L)	228	113	64
Neutrophils	1.8-7.7 (x10^9^/L)	13.8	2.7	1.8
Lymphocytes	1-4.8 (x10^9^/L)	0.6	0.2	0.4
Sodium	133-146 (mmol/L)	132	133	134
Potassium	3.5-5.3 (mmol/L)	3.9	3.5	3.7
Urea	2.5-7.8 (mmol/L)	6.5	4.8	5.1
Serum creatinine	59-104 (μmol/L)	105	73	80
Albumin	35-50 (g/L)	47	29	28
Total bilirubin	0-21 (μmol/L)	26	27	28
Alkaline Phosphatase (ALP)	30-130 (IU/L)	91	155	168
Alanine Aminotransferase (ALT)	0-41 (IU/L)	67	166	145
Gamma-glutamyl transferase (GGT)	0-71 (IU/L)	22	37	92
C-Reactive protein (CRP)	0-5 (mg/L)	157	x	231

Chest radiography was unremarkable. Three sets of blood cultures were positive for *Salmonella* Typhi. The patient was initiated on intravenous meropenem (2 g three times daily) and oral azithromycin (1 g once daily). The initial leukocytosis was followed by leukopenia on subsequent blood tests, which is a characteristic feature of typhoid fever.

**Figure 1:**
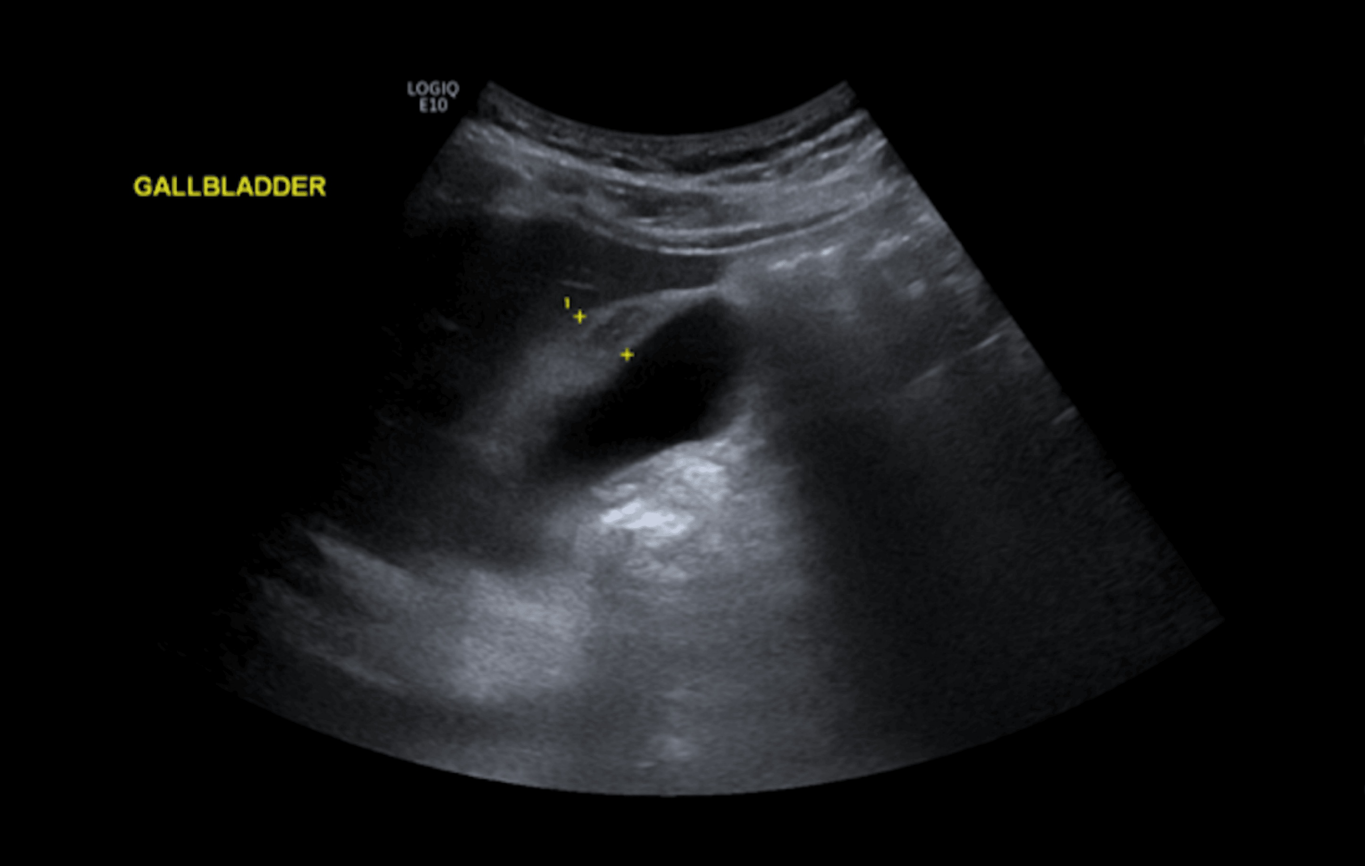
Ultrasound showing a focal thickening of the gallbladder, with no evidence of calculi, consistent with acalculous cholecystitis Gallbladder thickening of approximately 11 mm, as measured between the yellow crosses.

On the fourth day of admission, the patient remained febrile (39-40°C). Liver function tests worsened, with alanine aminotransferase (ALT) rising to 166 IU/L and alanine phosphatase (ALP) to 155 IU/L (Table 1), prompting further abdominal imaging. An abdominal ultrasound was performed to evaluate for additional gastrointestinal pathology, which revealed a focal gallbladder wall thickening measuring approximately 11 mm, in the absence of gallstones (Figure 1).

**Figure 2:**
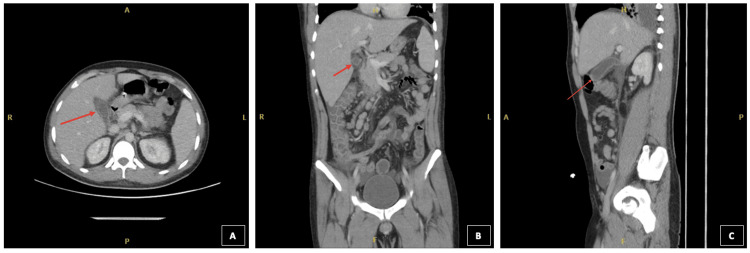
CT Thorax, Abdomen and Pelvis displaying a thick-walled gallbladder with surrounding pericholecystic fluid collection (as shown by red arrows) (A) Axial view; (B) Coronal view; (C) Sagittal view.

These findings were consistent with acalculous cholecystitis. A computed tomography (CT) scan of the thorax, abdomen, and pelvis subsequently demonstrated small bilateral pleural effusions and marked, oedematous gallbladder wall thickening (Figure 2).

On the seventh day of admission, the patient developed new oxygen requirements and was transferred to the intensive care unit. Chest radiography at this stage revealed new right lower zone consolidation, consistent with hospital-acquired pneumonia. Following clinical stabilisation, he was transferred back to the gastroenterology ward, where he completed a 14-day course of antibiotics and was subsequently discharged.

## Discussion

Acute acalculous cholecystitis is defined as inflammation of the gallbladder occurring in the absence of gallstones. It is most frequently observed in patients following major surgical procedures [7]; however, other etiological factors include trauma, burns, prolonged fasting, and infection. The underlying pathophysiology remains incompletely understood [8], although several risk factors have been implicated, including biliary stasis, starvation, gallbladder ischaemia, and endotoxin-mediated injury [8]. Importantly, the clinical presentation may differ from that of calculous cholecystitis [7]. While some patients may remain asymptomatic, others may present with right upper quadrant pain, fever, localised gallbladder tenderness, and leukocytosis [7].

Acute acalculous cholecystitis secondary to a primary bacterial infection is rare, particularly in adults [9]. A study conducted in Bangalore, India, reported that 24% of children with multidrug-resistant (MDR) typhoid fever developed acute acalculous cholecystitis [10]. Cases have also been documented in association with non-typhoidal salmonellosis and in individuals with acquired immune deficiency syndrome (AIDS) [9]. Following ingestion, *Salmonella* Typhi proliferates within the intestinal lumen and subsequently disseminates via the bloodstream and lymphatic system to the biliary tract or other infected organs [11]. Menendez et al. have shown that gallbladder epithelial cells are a site of in vivo replication for *Salmonella* species. This was shown to trigger a strong inflammatory response within the gallbladder epithelium, resulting in pro-inflammatory cytokine release and neutrophil infiltration [12]. 

The diagnosis of typhoid fever is typically established through the isolation of *Salmonella* species in blood cultures, as illustrated in this case. Although blood cultures are more likely to be positive in the early stages of disease, the yield is limited, with positivity rates reported in only 40-60% of patients [13]. Abdominal ultrasonography is the initial imaging modality of choice for suspected acute cholecystitis, with reported sensitivity ranging from 67-92% [13]. Major ultrasonographic criteria for acute acalculous cholecystitis include gallbladder wall thickening >3 mm, a striated gallbladder wall, a positive sonographic Murphy’s sign, pericholecystic fluid, and mucosal sloughing [14].

In this case, the patient’s ultrasound demonstrated a focal thickening of the gallbladder measuring 11 mm, without evidence of calculi. The ultrasound report did not describe the presence of oedema or pericholecystic fluid, necessitating further evaluation with a CT scan, which subsequently revealed an oedematous gallbladder wall. The diagnostic performance of ultrasound and CT in the assessment of acute cholecystitis has been extensively examined in the literature [14]. Although earlier studies had suggested that an ultrasound exhibits higher sensitivity than CT, Wertz et al. reported that, within their institution, CT demonstrated a significantly greater sensitivity (85%) for diagnosing acute cholecystitis compared to ultrasound (68%) [14]. The advantages of ultrasound should nonetheless be recognised, including its widespread availability, absence of ionising radiation, lower cost, and rapid acquisition of results [14]. These benefits are particularly pertinent in regions where typhoid fever is endemic.

Patients with a *Salmonella* Typhi infection of more than 10 days duration have been shown to be at approximately threefold higher risk of developing complications compared with those presenting earlier [15]. Among these, cholecystitis is particularly important, as ongoing inflammation can progress to ischaemia and necrosis, predisposing to gallbladder perforation, which has been reported in 2-11% of acute cholecystitis cases [16]. Although gallbladder perforation following acalculous cholecystitis in enteric fever is uncommon, the associated mortality is notably higher than that seen in calculous cholecystitis [17]. The recognition of this rare complication in typhoid fever is becoming increasingly relevant in the context of growing migration and international travel from endemic regions, alongside the emergence of more virulent and MDR *Salmonella* strains [18]. MDR *Salmonella* Typhi strains, resistant to traditional first-line agents such as ampicillin, chloramphenicol, and co-trimoxazole, with rising resistance to fluoroquinolones, pose a significant global health concern [19]. These factors underscore the clinical importance of considering cholecystitis and its sequelae when evaluating patients with prolonged or complicated courses of enteric fever, as illustrated in the present case.

In addition to the acute manifestations of typhoid fever, the chronic carrier state represents a significant public health concern. *Salmonella* Typhi has been shown to persist within the gallbladder, most frequently in association with gallstones, where the organism is capable of forming a protective biofilm [20]. As *Salmonella* Typhi is a human-restricted pathogen, chronic carriers contribute to ongoing transmission through the excretion of bacteria in stool and urine [20]. This carrier state is particularly problematic as the majority of affected individuals remain asymptomatic, lacking clinical features either during the acute illness or in the chronic phase, yet remain highly infectious [20]. Consequently, diagnosis is often challenging, complicating efforts to identify and manage this important reservoir of infection.

## Conclusions

This case demonstrates the rare occurrence of acute acalculous cholecystitis complicating *Salmonella* Typhi infection in an adult patient, a complication more frequently reported in paediatric populations. Its significance lies in highlighting an atypical but potentially serious manifestation of enteric fever, emphasising the need for heightened clinical awareness. This is particularly important in the context of increasing international travel and the emergence of multidrug-resistant strains. Prompt recognition is essential, as delayed diagnosis may lead to severe morbidity, including gallbladder perforation.

Heightened clinical vigilance is essential for patients with prolonged or complicated courses of typhoid fever, and timely imaging combined with multidisciplinary management can improve outcomes. The rising prevalence of MDR *Salmonella* Typhi further underscores the importance of antimicrobial stewardship, robust surveillance, and preventive public health measures, including vaccination and improved sanitation. In summary, this case highlights the importance of maintaining a high index of suspicion for atypical biliary complications in adult patients with enteric fever to ensure early diagnosis and appropriate treatment.
